# Diverticular disease is associated with an increased incidence rate of depression and anxiety disorders

**DOI:** 10.1007/s00384-021-03937-3

**Published:** 2021-05-03

**Authors:** Sven H. Loosen, Pia Paffenholz, Tom Luedde, Karel Kostev, Christoph Roderburg

**Affiliations:** 1 Clinic for Gastroenterology, Hepatology and Infectious Diseases, University Hospital Düsseldorf, Medical Faculty of Heinrich Heine University Düsseldorf, Moorenstraße 5, 40225 Düsseldorf, Germany; 2 Department of Urology, Uro-Oncology, Robot Assisted and Reconstructive Urologic Surgery, University Hospital Cologne, Cologne, Germany; 3 Epidemiology, IQVIA, Frankfurt, Germany

**Keywords:** Mood disorders, Diverticulitis, Major depression, Epidemiology, Antidepressants

## Abstract

**Background:**

Diverticular disease represents a gastrointestinal disorder of high prevalence in developed countries that often leads to psychological distress. Here, we aimed at evaluating a potential association between diverticular disease and depression or anxiety disorders in outpatients in Germany.

**Methods:**

Using the Disease Analyzer database featuring data of over 8 million patients treated in German general practices, we identified 61.556 patients with diverticular disease (ICD-10: K57) who were 1:1 matched by age, sex, index year, and the Charlson Comorbidity Index to 61.556 patients without diverticular disease. The association between diverticular disease and depression or anxiety disorders was evaluated in Cox regression models.

**Results:**

Within 5 years after the initial diagnosis of diverticular disease, 14.0% of patients with and 10.6% of individuals without diverticular disease were diagnosed with depression (HR 1.34, 95%CI 1.29–1.39, *p* < 0.001). Similarly, the incidence of anxiety disorder was significantly higher in patients with diverticular disease (HR 1.55, 95%CI 1.46–1.64, *p* < 0.001). Finally, the prescription rate for antidepressant drugs was significantly higher in diverticular disease patients compared to individuals without diverticular disease (9.4% vs. 6.1%, HR 1.56, 95%CI 1.49–1.62, *p* < 0.001). These associations were confirmed for different age groups and both sexes.

**Conclusion:**

Our data provide evidence that diverticular disease is associated with an increased incidence of depression and anxiety disorders. Despite that fact that confounding factors such as deprivation and patient personality have to be taken into account, we suggest that patients with diverticular disease are regularly screened for symptoms of depression and anxiety disorders.

## Introduction

Diverticular disease is a highly prevalent gastrointestinal disorder in developed countries with up to 60% of individuals affected by the age of 60 [1, 2]. Diverticulosis is defined as the appearance of sac-like protrusions (diverticula) along the gastrointestinal tract. Although its detailed etiology is only poorly understood, recent data suggest a polycausal genesis including environmental as well as lifestyle associated risk factors such as a diet low in fiber and high in red meat [3]. In addition, an impaired motility of the colon leads to the formation of diverticula. Due to a reduced passage velocity, there is an increase in intraluminal pressure, which can cause separation of the colonic lumen into chambers. Since the pressure is proportional to wall tension and inversely proportional to bowel radius, diverticula predominantly occur within the sigmoid colon. Finally, higher rates of diverticular disease have been described in obese patients as well as in smokers [4].

Despite being asymptomatic in many patients for years, diverticulosis and particularly its recurring complications such as diverticulitis represent chronic diseases that often lead to a high level of distress for these patients. As the imminent effects of chronic intestinal disease, such as inflammatory bowel disease, on aspects of psychological health have been substantially described in the past, an association of diverticular disease with mood disorders is very likely. In this line of thinking, it became increasingly evident that patients with diverticular disease should be managed by interprofessional teams [5]. However, although recent data suggest an association of diverticular disease with mental disorders such as dementia and anxiety [6, 7], only very limited systematic data on its mutual incidence exist to date.

In the present study, we aimed to evaluate a potential association between diverticular disease and mood disorders in outpatients treated in primary care practices in Germany between 2005 and 2018.

## Materials and methods

### Database

This study was based on data from the Disease Analyzer database (IQVIA), which compiles drug prescriptions, diagnoses, and basic medical and demographic data of over 8 million patients cases obtained directly and in anonymous format from computer systems used in the practices of general practitioners and specialists [8]. The database covers approximately 3% of all outpatient practices in Germany. Diagnoses (according to International Classification of Diseases, 10th revision [ICD-10]), prescriptions (according to Anatomical Therapeutic Chemical [ATC] Classification system), and the quality of reported data are being monitored by IQVIA. In Germany, the sampling methods used to select physicians' practices are appropriate for obtaining a representative database of general and specialized practices [8]. The “Disease Analyzer” database, used for analysis, contains anonymized electronic patient records. Patient data was analyzed in aggregated form without individual data being available. An individual consent form was not obtained following national and European legislation.

### Study population

**Fig. 1:**
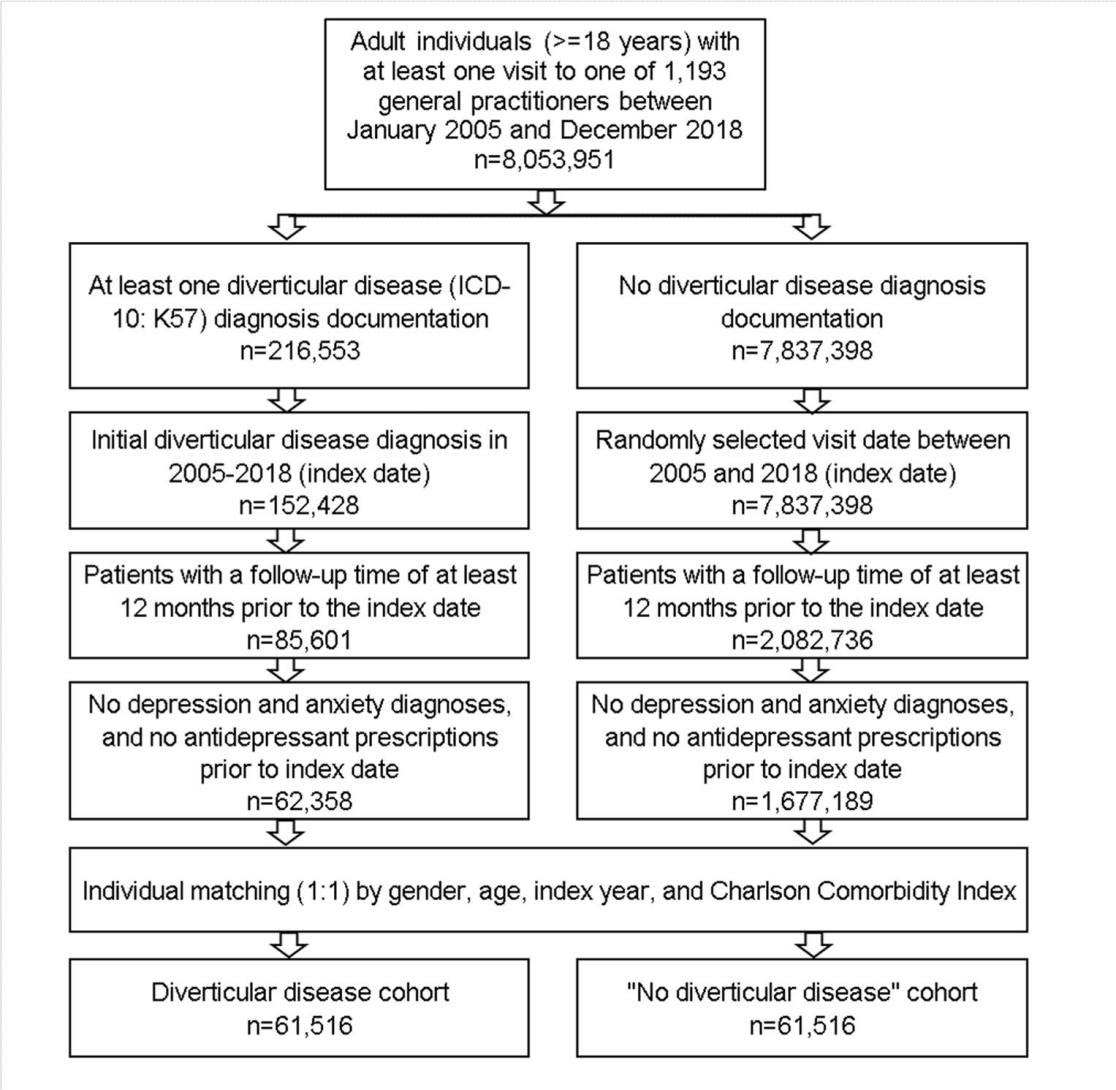
Selection of study patients

This retrospective cohort study included adult patients (≥18 years) with an initial diagnosis of diverticular disease (ICD-10: K57) in 1193 general practices in Germany between January 2005 and December 2018 (index date; Fig. 1). Further Inclusion criterium was an observation time of at least 12 months prior to the index date. Patients with depression (ICD-10: F32, F33), anxiety disorder diagnoses (ICD-10: F41), or antidepressant prescription (ATC: N06A) prior to index date were excluded.

Diverticular disease patients were matched to patients without diverticular disease by age, sex, index year, and Charlson Comorbidity Index (CCI). The Charlson index is a weighted index that accounts for the number and severity of comorbidities in administrative database studies and includes a wide range of comorbidities (macrovascular diseases, pulmonary diseases, gastrointestinal, liver and renal diseases, diabetes, tumors, and AIDS) (Quan et al. 2005). For patients without diverticular disease, the index date was that of a randomly selected visit between January 2005 and December 2018 (Fig. 1).

### Study outcomes and covariates

The main outcome of the study was the incidence of depression and anxiety disorder diagnoses as a function of diverticular disease. Furthermore, the incidence of antidepressant therapy defined as prescription of an antidepressant drug was analyzed.

### Statistical analyses

Differences in the sample characteristics between those with and those without diverticular disease were tested using chi-squared tests for categorical variables and Wilcoxon tests for continuous variables. The cumulative incidence of depression, anxiety disorder, and antidepressant therapy in the diverticular disease and non-diverticular disease cohorts was calculated for up to 5 years after the index date using Kaplan–Meier curves. Patients were censored at the time of first depression respective anxiety disorder diagnosis or on the day of the last diverticular disease record, or loss to follow-up, whichever occurred first. As no information on death was available, dead patients were considered as lost to follow-up in this study.

Cox regression models were conducted to study the association between the diverticular disease and depression, anxiety disorder, and antidepressant therapy incidence. These models were performed separately for five age groups, women and men. *P*-values were corrected using the Bonferroni adjustment method and were considered statistically significant at <0.006 (0.05 / 8 models). Analyses were carried out using SAS version 9.4 (SAS Institute, Cary, USA).

## Results

### Basic characteristics of study cohort

The present study included a total of 61,516 patients with diverticular disease and 61,516 patients without diverticular disease. The basic characteristics of the study cohort are summarized in Table 1. Mean age (SD) was 66.5 (13.7) years; 49.7% were women. The mean Charlson Comorbidity Index (CCI) was 1.4 (SD: 1.7) in both cohorts without significant difference.

**Table 1:** Basic characteristics of the study sample (after 1:1 matching by age, sex, index year, and CCI)

Variable	Proportion affected among patients with diverticular disease (%) *N* = 61,516	Proportion affected among patients without diverticular disease (%) *N* = 61,516	*p*-value
Age (Mean, SD)	66.5 (13.7)	66.5 (13.7)	1.000
Age 18–50	12.1	12.1	1.000
Age 51–60	19.2	19.2	
Age 61–70	25.3	25.3	
Age 71–80	28.8	28.8	
Age >80	14.6	14.6	
Women	49.7	49.7	1.000
Men	50.3	50.3	
Charlson Comorbidity Index excl. liver disease (Mean, SD)	1.4 (1.7)	1.4 (1.7)	1.000
CCI 0	38.7	38.7	1.000
CCI 1	25.8	25.8	
CCI 2	16.6	16.6	
CCI 3	8.9	8.9	
CCI >3	10.0	10.0	

Proportions of patients in % given, unless otherwise indicated

*SD* standard deviation

### Association of diverticular disease and depression

**Fig. 2:**
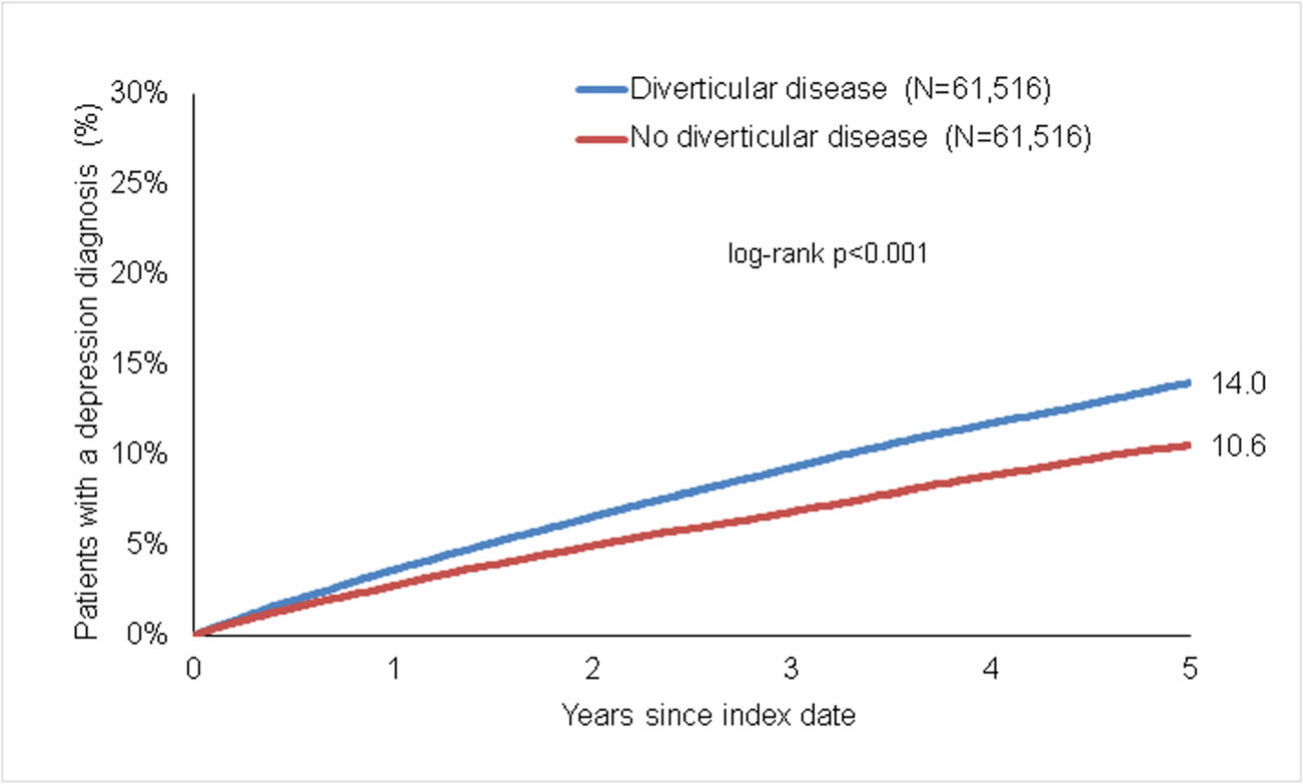
Kaplan-Meier curves for time to depression diagnosis in patients with and without diverticular disease

Within 5 years of the index date, 14.0% of patients with diverticular disease and 10.6% of individuals without diverticular disease were initially diagnosed with depression (log-rank *p* < 0.001) (Fig. 2). In regression analyses, diverticular disease was significantly associated with the incidence of depression (HR 1.34 (95% CI 1.29–1.39)). This association was descriptively slightly stronger in women than in men and was at strongest in the age group 18–50 years (Table 2).

**Table 2:** Association between diverticular disease and the incidence of depression, anxiety disorder, and antidepressant therapy in patients followed in general practices in Germany (Cox regression models)

	Depression		Anxiety disorder		Prescription of antidepressants	
Variable	HR (95% CI)	*p* value	HR (95% CI)	*p* value	HR (95% CI)	*p* value
Total	1.34 (1.29–1.39)	<0.001	1.55 (1.46–1.64)	<0.001	1.56 (1.49–1.62)	<0.001
Age 18–50	1.59 (1.45–1.74)	<0.001	1.68 (1.45–1.95)	<0.001	1.74 (1.55–1.96)	<0.001
Age 51–60	1.34 (1.25–1.44)	<0.001	1.58 (1.39–1.80)	<0.001	1.53 (1.40–1.69)	<0.001
Age 61–70	1.26 (1.17–1.35)	<0.001	1.40 (1.25–1.57)	<0.001	1.48 (1.35–1.63)	<0.001
Age 71–80	1.31 (1.23–1.40)	<0.001	1.61 (1.44–1.80)	<0.001	1.51 (1.40–1.64)	<0.001
Age >80	1.32 (1.21–1.45)	<0.001	1.64 (1.30–1.83)	<0.001	1.60 (1.41–1.80)	<0.001
Women	1.37 (1.32–1.44)	<0.001	1.58 (1.46–1.69)	<0.001	1.63 (1.54–1.73)	<0.001
Men	1.29 (1.23–1.36)	<0.001	1.52 (1.38–1.67)	<0.001	1.46 (1.36–1.56)	<0.001

### Association of diverticular disease and anxiety disorder

**Fig. 3:**
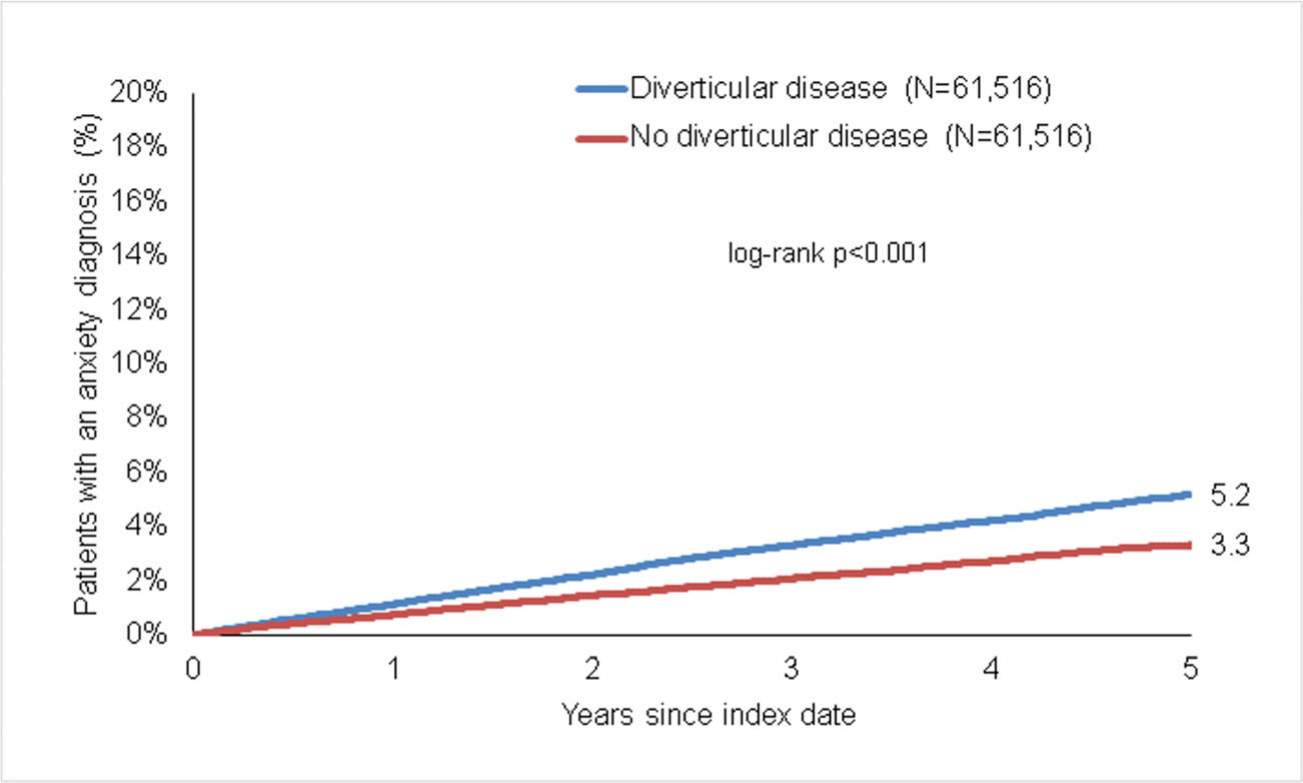
Kaplan-Meier curves for time to anxiety diagnosis in patients with and without diverticular disease

The incidence of anxiety disorder was 5.2% in diverticular disease and 3.3% in non-diverticular disease patients (*p* < 0.001) (Fig. 3). In regression analyses, diverticular disease was significantly associated with the anxiety disorder (HR 1.55 (95% CI 1.46–1.64)). This association was significant in both women and men as well as five age groups (Table 2).

### Association of diverticular disease and prescription of antidepressant drugs

**Fig. 4:**
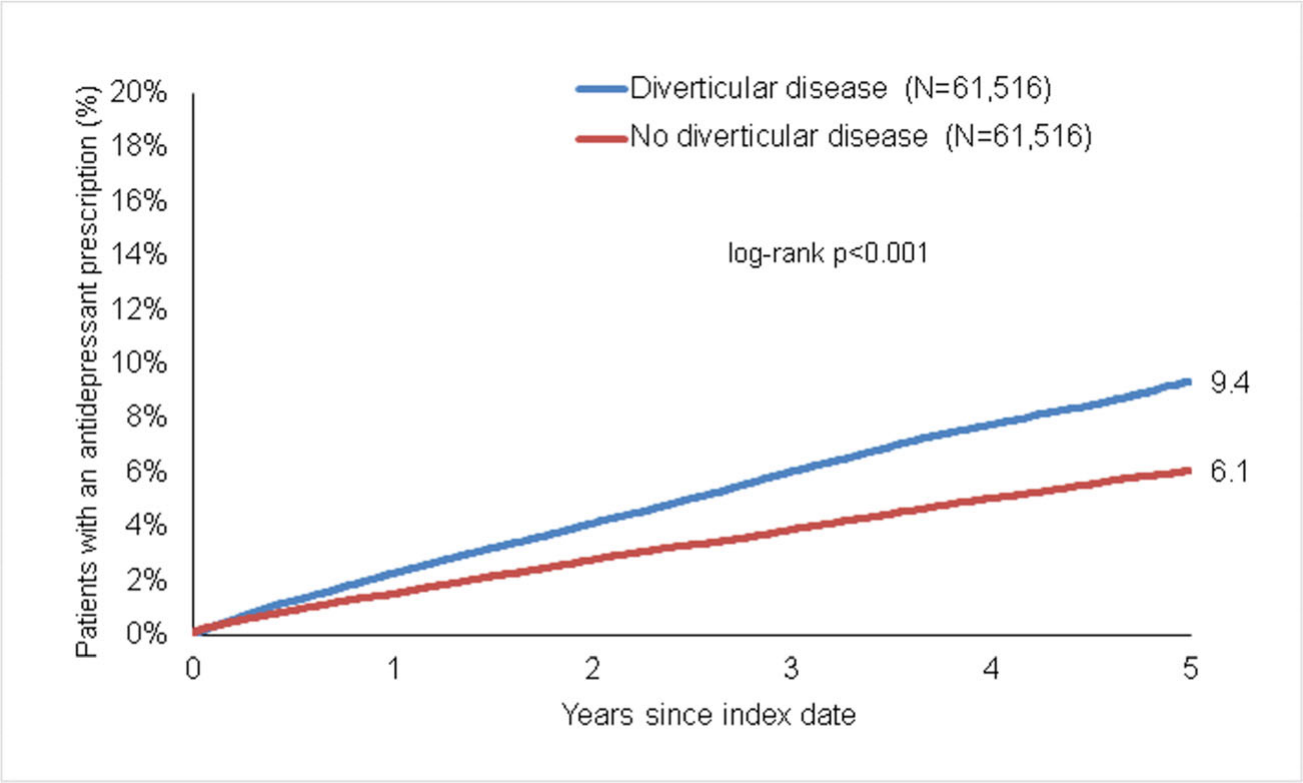
Kaplan-Meier curves for time to the first prescription of an antidepressant drug in patients with and without diverticular disease

Within 5 years of the index date, 9.4% of diverticular disease patients and 6.1% of non- diverticular disease patients received antidepressant prescription (*p* < 0.001) (Fig. 4). In regression analyses, diverticular disease was significantly associated with the antidepressant drug prescription (HR 1.56 (95% CI 1.49–1.62)). This association was stronger in women than in men. The strongest association was observed in patients aged 18–50, as observed for depression diagnosis (Table 2).

## Discussion

Diverticular disease represents a common gastrointestinal disorder in industrial countries implying an immense burden on patients’ quality of life, morbidity, and mortality. However, systematic analyses on the impact of diverticular disease on psychological aspects including depression and anxiety disorders are scarce and often inconclusive [9]. In the present study, we unraveled a significant association between diverticular disease and the incidence of depression as well as anxiety disorders in a well-defined cohort of 61,516 outpatients with diverticular disease treated in general practices in Germany between 2005 and 2018. In addition, diverticular disease was associated with a significantly increased prescription rate of antidepressant drugs.

Incidence rates of diverticulosis are rising globally, especially in developed countries in which about two-thirds of adults develop diverticular disease during lifetime [10]. Diverticular disease represents a chronic disease that, despite being asymptomatic in many patients, can lead to a wide range of clinical manifestations including common gastrointestinal symptoms such as cramping, bloating, flatulence, and irregular defecation but also more serious complications like diverticular bleeding or diverticulitis [3]. As only a minority of patients with a very severe course of disease are referred to surgery, which potentially can provide long-term relief [3], most patients are facing a protracted course of the disease that naturally involves a tremendous impact on psychological aspects as described for several other chronic conditions [11, 12]. Using the Disease Analyzer database, a well-established database of over 8,000,000 outpatient cases that was shown to be representative for the German ambulatory health sector [13], we aimed to evaluate the association between diverticular disease and mood disorders. Our study found a significantly increased incidence rate of depression in patients with diverticular disease within 5 years of the index date compared to patients without diverticular disease that were matched for important confounders such as age, sex, index year, and CCI. Similarly, the incidence of anxiety disorders and prescriptions of antidepressant drugs were significantly higher among patients with diverticular disease. Interestingly, this association was strongest in female patients and among patients between 18–50 years, which is in good agreement with the existing literature on sociographic analysis of depressive disorders [14]. Our results further align well with recent data showing a direct association between inflammatory bowel disease (IBD) and mood disorders such as anxiety or depression [15]. Given the extensive commonality of both diseases including chronic relapsing intestinal inflammation or the loss of intestinal barrier integrity [16], it is not surprising that mood disorders are not restricted to IBD but also occur in other chronic bowel conditions such as diverticular disease.

On a molecular level, the pathophysiological connection between intestinal disease and mood disorders remains not fully understood. The concept of the “gut-brain axis,” suggesting a functional association between the gut and the brain, has firstly been proclaimed in 1980 [17] and has gained increasing attention since. Gastrointestinal inflammation, as observed during acute and chronic diverticulitis, is often associated with microbial dysbiosis and leads to an impaired intestinal barrier function [18]. This condition results in a translocation of pro-inflammatory cytokines (e.g., TNF-a, IL-6), lipopolysaccharides (LPS) and microbial metabolites (e.g., short-chain fatty acids), which in turn crucially influence central neurotransmitter levels, the hypothalamic-pituitary-adrenal axis, and the brain barrier integrity. In this context, the toll-like receptor 4 (TLR4) and the brain-derived neurotrophic factor (BDNF) pathways have been suggested as key mediators in this bidirectional interaction [19, 20]. In clinical studies, an increased activity of the NLRP3 inflammasome has been observed in circulating immune cells of patients with major depression [21]. On a neurotransmitter level, low levels of tryptophan and serotonin were described as mediators of depressive symptoms and anxiety [21, 22]. In terms of intestinal dysbiosis, several clinical studies described increased levels of proteobacteria or prevotella and decreased levels of firmicutes and bacteroidetes in patients with major depression [23, 24]. Importantly, most of these pathological intestinal alterations have been associated with diverticular disease, suggesting a direct functional association between diverticular disease and mood disorders. Finally, these functional data are further corroborated by cerebral imaging data from patients with diverticulosis directly linking intestinal disease with structural brain alterations. In detail, Pitiot and colleagues observed decreases in gray matter density in the left and right dorsolateral prefrontal cortex, the mid-cingulate and motor cortex as well as increases in the left and right Brodmann Areas among patients with diverticular disease that correlated with patients’ symptoms [25]. Nevertheless, additional studies are warranted to further dissect the pathophysiological association between diverticular disease and depression or anxiety disorders.

We acknowledge some limitations of our study. First, all diagnoses in the Disease Analyzer database are recorded according to ICD-10 codes, which bears the potential risk of undercoding or misclassification of certain diagnoses. Second, the database does not provide systematic information on laboratory results and we were thus unable to evaluate whether chronic systemic inflammation might have been associated with an even higher incidence of mood disorders, which would further corroborate the “gut-brain axis” hypothesis. Third, the database did not allow drawing information on potential surgical treatment approaches in patients with diverticular disease that could be associated with a decreasing incidence of mood disorders, once the disease has been potentially cured. Fourth, there is the lack of data on lifestyle variables that might have an impact on the mood disorders (i.e., smoking status, alcohol use, physical activity, family status, employment). Fifth, there are no data from hospitals and gastroenterologist practices. Finally, there is the possibility that patient with diverticular disease develop a higher level of care-seeking that might in turn lead to higher detection and diagnosis rates of mood disorders.

Together, our data provide clear evidence that diverticular disease, representing one of the major gastrointestinal diagnoses in developed countries, is associated with an increased incidence of depression and anxiety disorders. These findings should trigger a higher awareness of mood disorders in this patient group, and we suggest that patients with diverticular disease are screened for symptoms of depression and anxiety disorders on a regular basis.
